# 

**Published:** 1891-04-18

**Authors:** 


					HOMAS'S HOSPITAL
IRWICH HOSPI TAL
.asooit Western Infirmary
WITH EXTRA* NURSING SUPPI.EMENT.
?%MISUKNt,&
h FOR INFANTS
T7
TSTlfbLAdd
AND INVALIDS.
No. 238. Vol; X.] Saturday,
April 18 th, 1891.
[One Tennt.

				

